# The Cutting Edge of Disease Modeling: Synergy of Induced Pluripotent Stem Cell Technology and Genetically Encoded Biosensors

**DOI:** 10.3390/biomedicines9080960

**Published:** 2021-08-05

**Authors:** Kamila R. Valetdinova, Tuyana B. Malankhanova, Suren M. Zakian, Sergey P. Medvedev

**Affiliations:** 1 The Federal Research Center Institute of Cytology and Genetics, The Siberian Branch of the Russian Academy of Sciences, 630090 Novosibirsk, Russia; valetdinova@bionet.nsc.ru (K.R.V.); malankhanova@bionet.nsc.ru (T.B.M.); zakian@bionet.nsc.ru (S.M.Z.); 2 E.N. Meshalkin National Medical Research Center, Ministry of Health of the Russian Federation, 630055 Novosibirsk, Russia; 3 Institute of Chemical Biology and Fundamental Medicine, The Siberian Branch of the Russian Academy of Sciences, 630090 Novosibirsk, Russia

**Keywords:** induced pluripotent stem cells, genetically encoded biosensors, genome editing, CRISPR/Cas9

## Abstract

The development of cell models of human diseases based on induced pluripotent stem cells (iPSCs) and a cell therapy approach based on differentiated iPSC derivatives has provided a powerful stimulus in modern biomedical research development. Moreover, it led to the creation of personalized regenerative medicine. Due to this, in the last decade, the pathological mechanisms of many monogenic diseases at the cell level have been revealed, and clinical trials of various cell products derived from iPSCs have begun. However, it is necessary to reach a qualitatively new level of research with cell models of diseases based on iPSCs for more efficient searching and testing of drugs. Biosensor technology has a great application prospect together with iPSCs. Biosensors enable researchers to monitor ions, molecules, enzyme activities, and channel conformation in live cells and use them in live imaging and drug screening. These probes facilitate the measurement of steady-state concentrations or activity levels and the observation and quantification of in vivo flux and kinetics. Real-time monitoring of drug action in a specific cellular compartment, organ, or tissue type; the ability to screen at the single-cell resolution; and the elimination of the false-positive results caused by low drug bioavailability that is not detected by in vitro testing methods are a few of the benefits of using biosensors in drug screening. Here, we discuss the possibilities of using biosensor technology in combination with cell models based on human iPSCs and gene editing systems. Furthermore, we focus on the current achievements and problems of using these methods.

## 1. Introduction

Many hereditary human diseases are hard to study due to a significant variety of pathological changes at the genome level and due to the multiplicity of metabolic pathways and functions of proteins in the whole body. Moreover, there is the impossibility of experimental studies on humans. Numerous experimental animal models do not adequately reproduce all genetic and phenotypic features characteristic of hereditary human diseases. Therefore, at present, the direction associated with the creation of in vitro models is actively developing. The developing technology of cell reprogramming to a pluripotent state promoted significant progress in this area [1]. The cells obtained in the work of Shinya Yamanaka were called induced pluripotent stem cells (iPSCs). In 2012, Yamanaka won the Nobel Prize for his outstanding scientific discovery. IPSCs possess practically all the properties of embryonic stem cells and, at the same time, are autologous to the somatic cell donor. IPSCs can be generated by reprogramming the somatic cells of any person, including from patients with hereditary diseases, using overexpression of only a few transcription factors. In this case, overexpression can be performed using methods such as transfection of episomal vectors, as well as the use of the Sendai virus, which ensures the intact state of the cell genome. Today, based on induced pluripotent stem cells, thousands of cell models have been created that cover diseases of almost all organs and systems of the human body. Most of the obtained cell lines are models of hereditary diseases. However, there are also models of multifactorial diseases that have a sporadic nature of occurrence. An especially urgent task today is the creation of cell models of neurodegenerative diseases, which are one of the crucial medical problems inherent in the “aging” population of people. However, to get closer to understanding the physiological, and most importantly, pathological processes occurring in the cell and the whole body, as well as to more effectively search and test methods for the treatment of various human diseases, it is necessary to reach a qualitatively new level of research of cell models of diseases based on iPSCs. In this case, the use of biosensors—molecules that can be used to measure practically any biological process occurring in a cell in normal or pathological conditions—has substantial prospects. The biosensor either changes its physicochemical characteristics or changes its localization by binding to any ligand. For example, the biosensor passes from the cytoplasm to the cell nucleus, or the biosensor changes its conformation due to covalent modifications triggered by the event under study. In this case, both a chemically synthesized dye directed to a specific cellular compartment and a protein that is produced from a genetic construct introduced into the genome of the object can act as a biosensor molecule.

## 2. IPSCs and Cell Models

Many drugs are currently undergoing initial testing in animals such as mice. However, this process can produce both false and negative results. For example, false positives include compounds that are likely to alleviate the disease phenotype in mice but do not benefit humans. This has been seen for creatine in the treatment of amyotrophic lateral sclerosis. It prolongs lifespan and maintains motor neuron function in mouse models but has not yielded measurable results in human clinical trials [2]. In addition, the toxicity of drugs differs depending on the animal species, so animal models may not be suitable for testing drug toxicity [3]. Although most clinical trials are expensive and time-consuming, preliminary testing in iPSC-based cell models can reduce costs and time, and disease modeling with iPSCs more accurately demonstrates drug efficacy and toxicity.

Cell models based on human iPSCs better reproduce the phenotype of diseases with early onset and worse than diseases that manifest in adults and the elderly [4]. The same problem arises when creating models of sporadic diseases (for example, sporadic forms of Alzheimer’s disease, amyotrophic lateral sclerosis), as well as polygenic diseases associated with more than one genetic cause in interaction with a combination of various provoking factors (for example, schizophrenia, autism spectrum disorders). However, the “aging” of cells in vitro can be performed by treatment with cellular stressors that target mitochondrial functions or pathways of protein degradation [5]. To obtain more complex models, the method of co-cultivation of more than one type of cells is used, including the technology of three-dimensional organoids obtained from iPSCs. The 3D organoids mimic the internal cellular organization and structure of human organs and reproduce the physiological conditions forming inside the organ. They have significant potential for studying the processes occurring inside a tissue or organ in response to the administration of medicinal compounds in a spatio-temporal context [6]. However, 3D cultivation technology still needs improvement. Optimization is needed for nutrient media and the composition of the extracellular matrix, which should provide conditions that most closely approximate the physiological characteristics of a person, with greater vascularization for effective growth, maturation, and supply of nutrients to cells.

**Figure 1:**
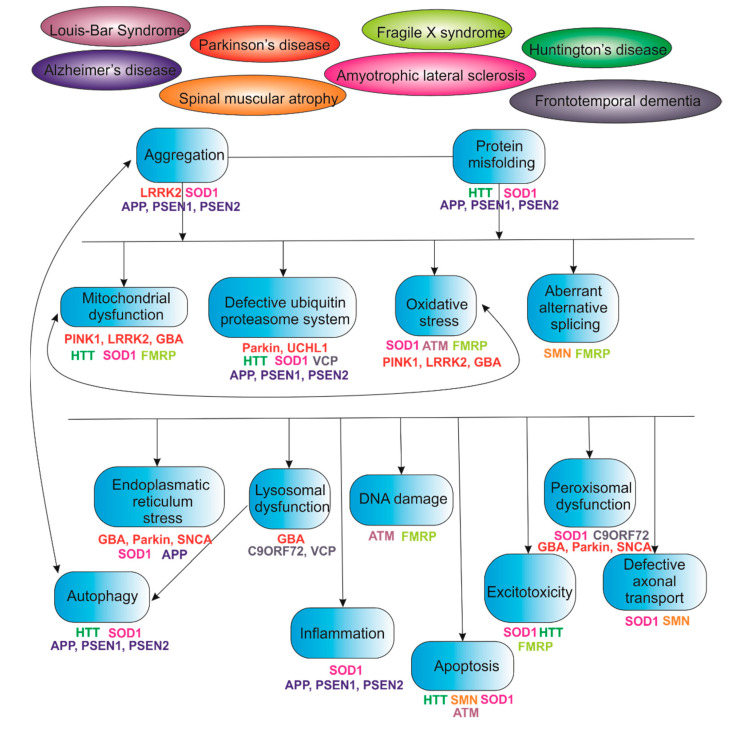
Universal pathological outputs in the hereditary diseases on the example of neurodegenerative diseases.

IPSCs have revolutionized personalized regenerative medicine and developmental biology. However, the etiology and pathophysiological mechanisms of many diseases remain unclear, especially for neurodegenerative diseases. Therefore, there is a need to create more complex cell models based on PSCs, and to use methods that would allow real-time monitoring and quantitative assessment of the dynamics of intracellular signals. One of these methods is using biosensors. Biosensors provide quantitative measurement of cellular processes, such as acetylation, methylation, cell cycle, cellular environment, cellular analytes, ions, kinases/phosphatases, oxidation/reduction, lipids, oxidative stress, ER stress, apoptosis, etc. A wide variety of biosensors allows for broad-profile screening of cellular processes. All this, in turn, makes it possible to study cellular processes in health and disease. As many hereditary and multifactorial diseases have common or interrelated pathological changes at the intracellular level, the use of biosensors in cell models based on human iPSCs opens up broad opportunities for deciphering the etiology and pathophysiological mechanisms of many diseases, significantly expanding our knowledge of human physiology at the cell level (Figure 1).

## 3. Biosensors Classification

**Figure 2:**
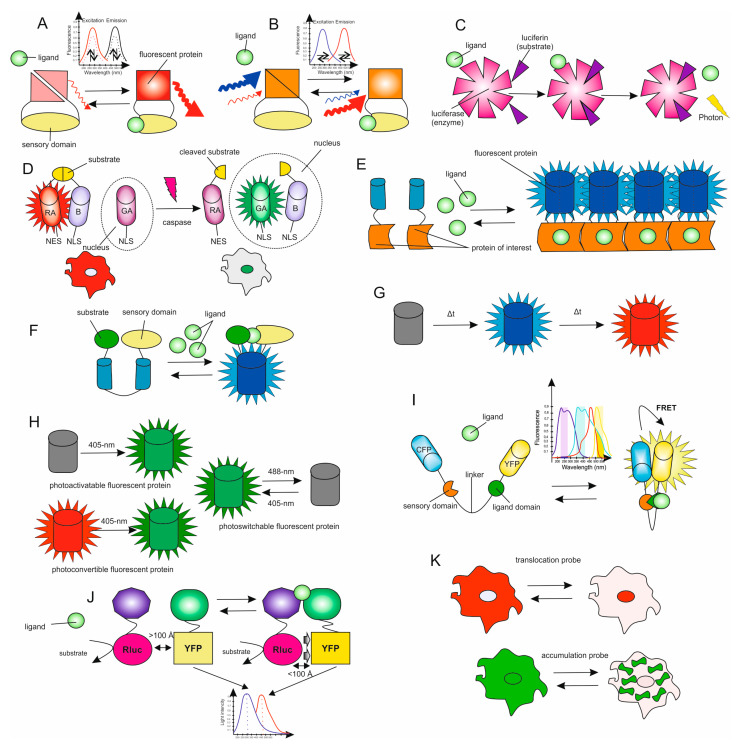
Biosensors classes. Rluc—*Renilla* luciferase, YFP—yellow fluorescent protein, CFP—cyan fluorescent protein, RA—red copy-A, GA—green copy-A, NLS—nuclear localization signal, NES—nuclear export signal. (**A**) Class I, type 1. Ligand-binding biosensors work on the principle of increasing or decreasing the fluorescence intensity. (**B**) Class I, type 2. Ligand binding to the chromophore or conformational modifications causes spectral shifts. (**C**) Class I, type 3. Ligand binding of biosensors is exhibited by bioluminescence. Binding of ligand to the chromophore causes an intramolecular reaction in which the cofactor (luciferin) is oxidized, leading to photon emission. (**D**) Class I, type 4. Acting dimerization-dependent fluorescent proteins on the example of intermolecular caspase-3 FPX biosensor. (**E**) Class I, type 5. BiFC biosensors. If the interaction occurs between two proteins of interest, this will facilitate the reconstitution of the fluorescent protein, forming the fluorescent complex. (**F**) Class I, type 6. Biosensors based on circularly permuted fluorescent proteins. (**G**) Class I, type 7. Fluorescent timers. (**H**) Class I, type 8. Biosensors based on molecular photoswitches. (**I**) Class II, type 1. FRET-based biosensors. Includes probes where ligand binding changes the FRET efficiency between the two chromophores. (**J**) Class II, type 2. BRET biosensors, the donor chromophore is a chemiluminescent protein and the acceptor is fluorescent protein. (**K**) Class III, translocation- or accumulation-based biosensors.

All biosensors can be divided into three large classes (Figure 2). The first class includes biosensors, the functioning of which is based on modifications of the spectral properties or quantum yield of the chromophore due to a change in the ligand concentration. The second class is based on the Förster resonance energy transfer (FRET), and the third on the movement or accumulation of a fluorophore.

Biosensors of the first class can be divided into eight types: the first type works on the principle of increasing or decreasing the fluorescence intensity [7,8,9,10] (Figure 2A), the second type is ratiometric when binding with a ligand, and the peaks of excitation and/or emission of the fluorophore shift [11,12,13,14] (Figure 2B). The second type of biosensor is easier to calibrate, since it is independent of the inhomogeneity of the fluorophore loading, photobleaching, or fluorophore leakage. In addition, for this type of biosensor, it is possible to correct changes in the focal plane of the image or motion artifacts. In many ways, a fluorescent biosensor is an ideal tool for monitoring signaling pathways within cells. The absorption and emission of light by a fluorophore occurs within nanoseconds, and it can be accurately localized in space, since the emitted wavelength is smaller than many cellular structures. Thus, the data obtained using fluorescent biosensors make it possible to describe with high accuracy the extremely fast processes occurring in the cell and intracellular structures. When using sophisticated and sensitive microscopic equipment, fluorescent biosensors are perfect tools for visualization of intracellular processes in vivo, and it is possible to obtain data that could not be obtained using traditional biochemical methods.

The third type includes luminescent biosensors (Figure 2C). In chemiluminescence, ligand binding causes an intramolecular reaction in which luciferin is oxidized, resulting in the emission of photons. The photon emission rate is proportional to the amount of bound ligand. The signal received from a luminescent biosensor is weaker than the corresponding fluorescence signals, and highly sensitive equipment is required for its detection. However, in the case of using luminescent biosensors, there is practically no background glow as compared to fluorescent biosensors, when a high background of cellular autofluorescence is often observed. Consequently, luminescent biosensors have a better signal-to-noise ratio, and, accordingly, are a more suitable choice for high-throughput screening studies and in vivo experiments [15,16].

The fourth type is dimerization-dependent fluorescent proteins (ddFPs). This system is composed of two copies of fluorescent proteins as a pair [17]. One copy in the ddFP pair contains a chromophore (copy-A), while the other copy does not (copy-B). When they exist separately, the fluorescence of copy-A is dim; however, the ddFP system becomes bright when copy-A and copy-B form heterodimers. Thus, we can detect protein–protein interactions by measuring the increase in the fluorescence brightness of the ddFP system. For example, the intermolecular caspase-3 FPX biosensor is composed of two parts. The first part contains red copy-A (RA)-NES and copy-B-NLS, connected with the caspase-3 substrate sequence DEVD; the second part is green copy-A (GA)-NLS [13] (Figure 2D). In the default state, the heterodimer of RA and the copy-B display red fluorescence in the cytosol. Upon caspase-3 activation, the substrate DEVD between RA and B is cleaved, and the released B-NLS can translocate into the nucleus, where it binds to GA-NLS and displays green fluorescence. Therefore, the activity of caspase-3 can be monitored by the FPX-mediated changes in fluorescent colors at subcellular locations.

The fifth type includes biosensors based on bimolecular fluorescent complementation (BiFC) (Figure 2E) [18]. In this case, the polypeptide chain of the fluorescent protein is divided into two non-fluorescent portions, which, when combined, form a functional fluorescent protein. This “split” fluorescent protein has a variety of applications. For example, split variants of fluorescent proteins are useful for the detection of protein–protein interactions due to their inability for spontaneous reassociation in a cell. In this case, researchers create chimeric proteins in which the first of the studied pair of target proteins is attached to one part of the fluorescent protein and the second to another. If the target proteins interact with each other, the adjacent parts of the fluorescent protein combine and the cell generates a fluorescent signal. If there is no interaction, the fluorescent protein does not reassociate and does not fluoresce.

The sixth type includes biosensors based on circularly permuted fluorescent proteins. As the N- and C-termini of a fluorescent protein are located in the same direction, they can be connected with a linker, creating new termini near the chromophore [19]. In this circularly permuted FP (cpFP), the new termini can be further fused to the sensing domains whose conformational rearrangement can modulate the fluorescent intensity of cpFP (Figure 2F). Compared with the original fluorescent protein, the cpFP displays lower fluorescence intensity due to its relatively weak folding near chromophores. Thus, the conformational rearrangement of the sensing domains can enhance the brightness of cpFP. Various biosensors have been developed based on cpFP: Ca^2+^ sensors [20,21], cpFP-based sensors to detect cofactors [22], cAMP [23], ATP [24,25], or neurotransmitters such as glutamate and GABA [26,27].

The seventh type includes biosensors based on fluorescent timers (Figure 2G). The first fluorescent timer was developed with DsRed-E5, a DsRed mutant with two substitutions V105A and S197T [28]. In particular, S197T is suggested to directly contact the chromophore as an analogue of T203 in GFP, enabling the mutant E5 to exhibit a green intermediate fluorescence before its full maturation to red fluorescence. The green-to-red color conversion of DsRed-E5 is time dependent. Thus, this unique fluorescent protein can be utilized as a fluorescent timer to sense the relative ages of the attached proteins of interest.

The eighth type includes biosensors based on molecular photoswitches [29,30] (Figure 2H). Irradiation with an exact wavelength causes a change in the spectral properties of the photoswitch, which can be reversible or irreversible, which makes it possible to visualize the signal with high spatial and temporal resolution. Such biosensors can be classified as photoactivated if they increase their radiation intensity upon stimulation, and photoconvertible if they change their radiation spectrum upon exposure. The ability to activate or transform a small subset of molecules makes these biosensors a fascinating and innovative tool for ultra-high-resolution imaging, and for pulse-chase experiments for tracking organelle movement, and in studying protein–protein interactions [31]. However, their fluorescence is often very dim, they often require photoactivation or conversion of UV radiation, and the conversion of green light to red covers the radiation spectrum in which it is difficult or impossible to obtain two-color images.

The second class of biosensors allows the assessment of FRET between two fluorescent molecules (most often two GFP mutants) that are covalently linked to the sensory domain or domains (Figure 2I). In these sensors, ligand binding or their covalent modification (for example, phosphorylation) does not change the fluorescent characteristics of chromophores, but changes the distance between them and/or their orientation and, consequently, the FRET efficiency. This is expressed as a change in the ratio between the intensity of light emitted by the donor and acceptor chromophores (upon excitation of the donor), respectively, as these sensors are ratiometric [32]. In some cases, the efficiency of FRET and its changes are monitored by measuring the lifetime of an acceptor or donor molecule using time-resolved fluorescence microscopy (FLIM) [33]. In a particular case of resonant energy transfer, called bioluminescent resonant energy transfer (BRET), the donor chromophore is a chemiluminescent protein, luciferase (or one of its variants), and the acceptor is YFP (Figure 2J). The relatively low intensity of BRET signals is an important limitation in the analysis of single cells [34,35].

The third class of biosensors makes it possible to track the movement of a fluorescent signal between cellular compartments (Figure 2K). For example, biosensors sensitive to membrane potential accumulate in mitochondria; weak bases accumulate in acidic organelles under the action of ΔpH [36,37]. These “moving sensors” are usually used for qualitative measurements because they are difficult to use for obtaining quantitative information.

## 4. Chemical Biosensors

Chemical biosensors are often organic fluorescent dyes—a class of organic molecules that contain a fluorescent core framework with a massive conjugate system and some auxochromes or active groups (such as carboxyl, amino, amide). The framework of the fluorescent core allows them to absorb specific excitation light and emit it as fluorescence. An auxochrome or active group is capable of changing wavelengths and enhancing fluorescence to recognize different biomolecules. Now, there are many types of organic fluorescent dyes which allow measuring the levels of various ions, metabolites, etc. [38].

For example, commercial dyes Fura-2, Fluo-4, Fluo-8 are used to determine intracellular Ca^2+^ [39]. The main advantage of chemical indicators over genetically encoded sensors is the wide range of affinities for Ca^2+^. Additionally, it is easy to administrate and quickly use these dyes for experiments. The protocols for the use of chemical indicators Ca^2+^ are well documented. The main disadvantages are that the cellular localization of Ca^2+^ indicators is difficult to control, and there are difficulties in targeting a specific organelle. In addition, chemical indicators tend to degrade and are ultimately displaced from the cell during long-term experiments [40]. To solve the problem of compartmentalization, researchers created indicators with a large dextran label [41]. This allows the recording of Ca^2+^ levels over long periods, up to several days [42]. However, the disadvantage of dextran-labeled dyes is that they are more difficult to deliver into cells.

The most widely used chemical pH sensors are BCECF [43] and carboxy-SNARF-1 [44], as they have desirable photophysical properties for near-neutral intracellular H^+^ concentration determination. Fluorescein and fluorescein derivatives such as carboxyfluorescein are common indicators of pH [45], but they quickly seep out of the cytosol through cell membranes and lead to inaccurate pH measurements. HPTS, another widely used chemical sensor for determining intracellular pH, tends to be retained within living cells because it has three sulfonate groups and can be used to measure acidic and near-neutral pH values [46]. However, HPTS cannot permeate into cells, and additional modifications are required for its delivery.

There are also chemical sensors that are sensitive to changes in membrane potential, which have fast and slow responses. While the slow dye responds to a change in membrane potential with a more pronounced alteration in fluorescence, the fast dye responds quickly enough to detect action potentials in neurons. Among the rapid response group, chemical sensors of the amino-naphthyl ethenyl pyridinium (ANEP) series are the most reliable and sensitive for detecting submillisecond changes in membrane potential in various tissues, cells, and model membrane systems [47,48].

Indo- (DiI), thio- (DiS), and oxo- (DiO) carbocyanines with short alkyl tails (<7 carbon atoms) were among the first developed potentiometric fluorescence sensors [49]. These cationic dyes can accumulate on hyperpolarized membranes and migrate to the lipid bilayer. Accumulation on the inner membrane usually leads to a fluorescence decrease, although the magnitude and even the direction of the fluorescent signal strongly depend on the concentration of the dye and its structural characteristics. To obtain a specific mitochondrial signal and exclude a potentially independent background due to staining of the endoplasmic reticulum and other intracellular membranes, low concentrations (<100 nM) of such dyes are required. A usual indicator of mitochondrial activity is rhodamine 123 [50]. TMRM and TMRE, methyl and ethyl esters of tetramethylrhodamine, are closely related to rhodamine 123. As with rhodamine 123, the accumulation of these cationic dyes in mitochondria results in a decreased fluorescence signal due to self-quenching. However, TMRM and TMRE penetrate the plasma membrane faster than rhodamine 123, and a higher level of fluorescence signal allows the use of even low concentrations of this type of sensor, which avoids aggregation. In a high-throughput drug screening that affects the mitochondrial membrane potential in living cells, researchers successfully used TMRE [51].

## 5. Genetically Encoded Biosensors

The first genetically encoded biosensor, Cameleon, was developed by Californian scientists in 1999. It was based on a green fluorescent protein variant and was used to visualize the level of calcium ions in living cells [52]. Currently, the Biosensor Database (https://biosensordb.ucsd.edu/; accessed on 14 April 2021) contains more than 800 genetically encoded biosensors that allow to study various cellular and extracellular processes.

The combination of iPSCs with genetically encoded biosensors is an incredible breakthrough in studying the molecular processes of human diseases. Pharmaceutical companies can use cells with biosensors to screen new medicinal compounds and even entire libraries for toxicological studies. Genetically encoded biosensors allow visualizing the effect of a compound on cells, as well as a quantitative assessment of this effect.

Typically, genetically encoded biosensors are delivered into cells by transient transfection of plasmid vectors expressing the biosensor. The disadvantage of this method is the problem of the molar number of the biosensor in the cell. It is practically impossible to accurately determine the number of molecules of plasmids expressing the biosensor that entered in the each cell during a given transfection. This leads to problems in the measurement of the intensity of the signal from the biosensor while using ratiometric biosensors. Another way is to generate transgenic cells with constitutive or controlled expression of a biosensor by the transduction of cells with viruses. However, the introduction of transgenes in this way is also impossible to control. It is impossible to know exactly how many copies of the transgene are introduced into the cell or the amount of biosensor in the individual cell. In addition, there is the problem of insertional mutagenesis, which can significantly contribute to the normal functioning of cells and the functioning of the biosensor.

The solution to this problem is the directed introduction of transgenes into the genome of cells using homologous recombination. The development of tools for targeted and efficient gene editing made possible efficient homologous recombination in the selected locus. One of the most effective tools is the CRISPR/Cas9 system [53]. This system has high efficiency and specificity, but, at the same time, it is simply used to create genetic constructs. It is enough to place a synthetic DNA fragment of about 20 nucleotides in length (spacer) into a vector expressing noncoding RNA (sgRNA) and Cas9 nuclease to create a construct that recognizes a specific DNA sequence in the genome. The spacer is part of the sgRNA, and its sequence determines the binding specificity of the sgRNA to the target DNA (protospacer). At the same time, Cas9 nuclease complexed with sgRNA introduces a double-stranded DNA break. The DNA breaks stimulate the homologous recombination process. For homologous recombination, a donor DNA molecule is required, which can be oligonucleotides or plasmid constructs.

Safe-harbor loci are the places in the genome for the “safe” introduction of transgenes. Moreover, the expression of transgenes occurs without disrupting the expression of adjacent or more distant genes. In addition, these loci are epigenetically available, which provides the stable expression of transgenes. There are several safe-harbor loci in the human genome. The most widely used human safe-harbor locus is *AAVS1* on chromosome 19q, which was originally identified as a site for recurrent insertion of the adeno-associated virus [54]. Other potential safe-harbor loci are *hROSA26* [55] and the *CCR5* chemokine receptor gene, which, when disrupted, provides resistance to human immunodeficiency virus infection [56]. In addition, research is ongoing to find new loci. In 2019, work was reported, where the authors discovered 35 more new potential safe-harbor loci [57]. This allows the combination of various transgenes, including biosensors, in one cell genome.

### 5.1. Biosensor Expression Regulation

**Figure 3:**
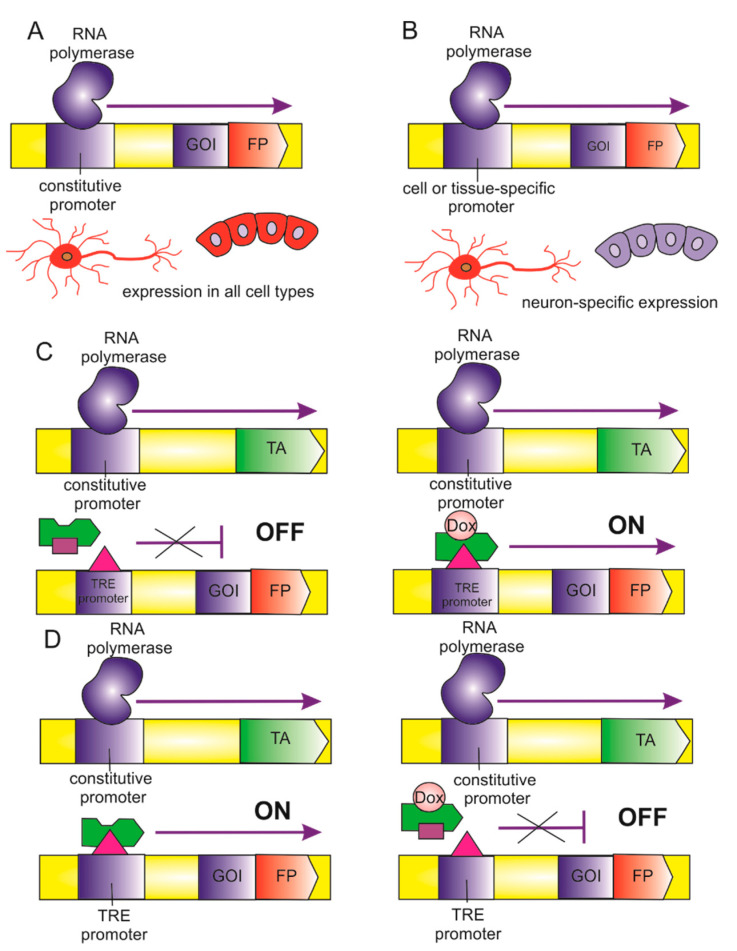
Expression of genetically encoded biosensors. TA—transcriptional activator, TRE—tetracycline responsive element, FP—fluorescent protein, GOI—gene of interest, Dox—doxycycline. (**A**) Constitutive promoter. (**B**) Tissue-specific promoter. (**C**) Doxycycline-inducible expression, the Tet-On system. (**D**) Doxycycline-inducible expression, the Tet-Off system.

In addition to the controlled copy number of the transgene, a second strategy for the “smart” use of biosensors is the use of various promoters for the transgene expression (Figure 3). The standard way is to use a constitutive promoter (Figure 3A). The constitutive promoter is, of course, very convenient and ensures stable expression of the transgene. However, the constant high expression of the transgene and the high capacity of the “foreign protein” may contribute to the state of the cells.

Another way is to use a tissue-specific promoter (Figure 3B). The biosensor transgene can be inserted into the genome under the control of a tissue-specific promoter. A tissue-specific promoter is a special case of a constitutive promoter, but its expression depends only on the cell type. IPSC differentiation protocols do not give 100% yield of the target cell type. Usually, there is a mixed population of different cell types. Using a tissue-specific promoter allows for the disposal of the biosensor signal from unwanted cells. This, in turn, leads to obtaining results only from a specific target cell type. On the other hand, a fragment of a tissue-specific promoter can be introduced into the safe-harbor locus. For example, a fragment of the CD43 promoter was used for the expression of a reporter fluorescent gene at the *AAVS1* locus [58]. A fragment of the CD43 promoter provided tissue-specific controlled expression of the transgene in the cells of hematopoietic clones. However, tissue-specific promoters and even their fragments can be so extended that it is difficult to pack them into a genetic construct. In addition, the expression of some genes is driven by enhancers. This, in turn, also complicates the search for the most suitable promoter for the expression of the transgenes.

An alternative strategy may be introducing the transgene sequence at the 5′- or 3′-end of the tissue-specific gene. Moreover, in the case of insertion of the transgene into the 3’-end of the gene, it will be necessary to remove the stop codon. The transgene can be fused to a tissue-specific protein, for example, through the Fusion motif, or it can be separated through a 2A peptide or IRES element. In this case, the level of the target gene expression must be high for the detection of a fluorescent biosensor. Therefore, it is critical to consider the expression level of the selected gene. There is a TiProD database to select specific promoters. It is a database of human promoter sequences, where one can find individual promoters and the expression pattern that they mediate, and extract sets of promoters by their tissue-specific activity. The database is available at http://tiprod.bioinf.med.uni-goettingen.de (accessed on 20 April 2021).

Finally, perhaps using an inducible promoter is the most optimal for mammalian cells, which are supported and controlled by very complex genetic networks, and fine-tuning of genetically encoded biosensors expression is required. Moreover, irreversible manipulations of gene expression can often induce compensatory responses [59,60]. Therefore, the ability to turn on and off the expression of transgenes makes it possible to exclude side and off-target effects. Currently, several inducible gene systems can be used depending on the experimental requirements and designs. There are tetracycline-controlled operator systems, cumate-controlled operator systems, a chimeric system based on protein–protein interaction, systems induced by light of a certain wavelength, a tamoxifen-controlled recombinase system, and finally, an expression system with a regulated riboswitch [61]. In cultured human cells, tetracycline/cumate-controlled systems are most attractive because of their ease of use, high efficiency, and few side effects. The Tet-On system is the highest priority due to its low leakage (Figure 3C). The Tet-Off system is preferred in experiments where the presence of tetracycline in the culture medium must be avoided (Figure 3D). However, the disadvantage of inducible expression of the biosensor, as well as in the use of a constitutive promoter, is that the biosensor is expressed in all cells—in target and non-target cell types. In this situation, it is necessary to select the most optimal protocol for iPSC differentiation with the maximum yield of the target cell population. Finally, another way to remove a non-target cell population is sorting by a specific surface marker [62].

### 5.2. Combination of Biosensors

**Figure 4:**
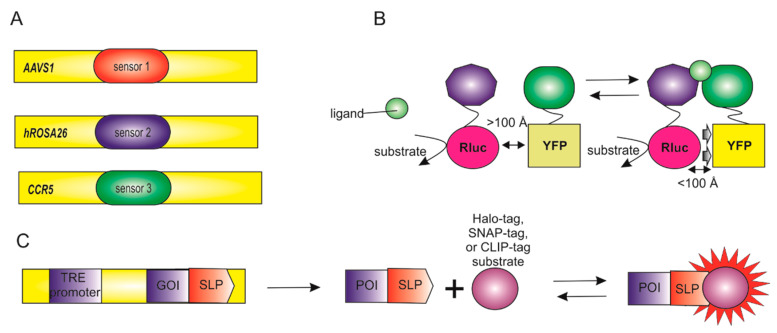
Combination of biosensors. (**A**) Combination of several biosensors at different safe-harbor loci. (**B**) Combination of different biosensors types, for example, chemiluminescent protein and the fluorescent protein. BRET. (**C**) Combination of organic dyes and a genetically encoded protein. SNAP-tag, Halo-tag, and CLIP-tag are composed of a small protein that mediates the covalent binding between the genetically encoded target and the fluorophore.

The variety of biosensors and the presence in the genome of several loci that are safe for integration make it possible to combine several biosensors in one genome (Figure 4A). On the one hand, one can study several different processes in one cell. It allows studying everything on the same genetic background, which avoids the influence of polymorphisms. On the other hand, one can use a sensor of the same process, for example, the redox potential of glutathione, but with a different cellular localization or type of biosensor—fluorescent and non-fluorescent.

While using two fluorescent biosensors, the excitation wavelengths of the fluorophores must be different. This is critical because if the excitation wavelengths overlap, then the signal from the two biosensors will overlap and add up.

The second option is a combination of a fluorescent and chemiluminescent biosensor (Figure 4B). In this case, it can be two separate biosensors, or a combination in one, for example, BRET sensors. In this case, energy from the fluorescent protein is transferred to the chemiluminescent protein. Tet-On/Tet-Off systems can be used in combination. When tetracycline is added to the medium of the cells, the expression of one biosensor stops, and the expression of the second starts.

Finally, there are some biosensors that combine organic dyes and a genetically encoded protein (or peptide) (Figure 4C): the tetracysteine/biomarsenic system and its variants (FlAsH and ReAsH), based on high-affinity interactions between trivalent arsenic compounds and a short peptide sequence containing pairs of closely spaced thiols [63]; fluorogen-activated proteins (FAPs) obtained from single-chain antibodies capable of binding an organic dye [64,65]; SNAP-tag, Halo-tag, and CLIP-tag consisting of a small protein (such as hAGT and halogendehalogenase) that mediates covalent binding between a genetically encoded target and a fluorophore [66,67,68].

### 5.3. Biosensor Selection

Given the variety of biosensors available, both chemical and genetically encoded, the choice of the optimal sensor requires a careful assessment of their biophysical properties which are more in line with the needs of the experimenter. The most important parameters to consider when choosing a biosensor are the following:

1. Ease of use. One of the critical factors when choosing a biosensor is the availability of the equipment needed for the measurements. For example, ratiometric biosensors based on FRET require sophisticated microscopic equipment to quickly (or simultaneously) collect data from two or more fluorescence channels. At the same time, for biosensors that measure fluorescence intensity, uninvolved instruments are needed to collect data. It is also necessary to consider the availability of the materials used. Chemically encoded biosensors must be constantly purchased, while genetically encoded sensors are infinitely renewable. In addition, some staining protocols using chemically encoded biosensors are cumbersome, especially if the biosensor is impermeable to cells. Finally, many luminescent biosensors require an additional step of adding a substrate such as coelenterazine [69].
2. Consideration of the spectral features of the fluorophore. The spectral properties of the biosensors should be carefully analyzed to obtain two-color images and avoid crosstalk or fluorophore leaks; red-shifted excitation wavelengths are preferred for in vivo experiments due to their deep tissue permeability.
3. Signal-to-noise ratio. This ratio depends on changes in fluorescence caused by ligand binding compared to fluctuations recorded by autofluorescence. To minimize photobleaching, researchers use the lowest excitation intensity and/or exposure time required to achieve an acceptable signal-to-noise ratio. For a 16-bit camera, a good rule of thumb is that the lowest signal is at least 1000 samples above the background.
4. Sensitivity. One of the indicators of the sensitivity of a biosensor is the dynamic range, which is the ratio between the minimum and maximum values that the biosensor can detect. The sensitivity of the biosensor used is application specific. For example, to measure constant concentrations of a molecule or ion, it is recommended to choose a sensor with a dissociation constant (Kd) equal to or close to the expected concentration. An even more rigorous approach is to measure the concentration at rest using several biosensors, each of which has a slightly different Kd value. If the goal is to measure a change in concentration or activity when the signal is expected to be weak, then the biosensor with the highest dynamic range and Kd value, at which the concentration change will be within 5 Kd, will be most sensitive.
5. Kinetics. The rate of binding/detachment of the ligand with the biosensor is crucial, which determines the ability or inability of the sensor to report rapid kinetics, as well as selectivity for the ligand, that is, the specificity of ligand binding in comparison with other molecules, and the sensor’s affinity for the ligand. The in situ environment can significantly affect the Kd and hence any quantitative analysis in living cells. This problem applies to any sensor and, in particular, to target sensors that are localized in subcellular niches, the environment of which can vary in terms of pH, viscosity, the concentration of heavy metals, and binding to local proteins. Moreover, the addition of the target peptide itself can affect the properties of the sensor. Therefore, accurate measurement of the Kd value is extremely important if any quantification is needed. As mentioned above, ratiometric sensors are easier to calibrate than sensors that only change signal strength. In the latter case, the information is usually qualitative rather than quantitative. Since cellular signals occur at different time scales, the kinetics of the process under study will also determine the optimal sensor to use. Small molecules often offer higher on and off rates (kon and koff) and thus give better resolution for events that occur on fast time scales. As a general rule of thumb, the kinetics of the sensor must accurately reproduce the biological signal. However, the slow turn-on may have some advantages in detecting low, weak signals.
6. Signal localization. Genetically encoded sensors can easily target specific organelles or cell compartments by triggering localization signals. In the case of using chemical sensors, the task becomes more complicated. It is crucial to understand that the environment (pH, redox state) of a given organelle can limit the available sensors. For example, endocytic vesicles maintain an acidic pH (<6), which quenches YFP fluorescence and thus inhibits the use of CFP-YFP sensors in such acidic compartments.
7. Quantification. For events requiring quantification, ratiometric biosensors or FRET sensors are generally preferred over fluorescence intensity sensors. FRET-based biosensors usually have a wider dynamic range. However, the fluorescence intensity is largely determined by the target molecule itself, as well as the level of sensor expression in a cell or organelle. Consequently, this type of biosensor is poorly suited for quantifying the concentration of an investigated molecule or changes in concentration. Calibration is needed to perform a comparative analysis of the data obtained using a particular biosensor in different types of cells or under the influence of various stimuli. It is necessary to calibrate the biosensor to measure the minimum and maximum signal levels. Although individual signal levels may vary from one cell to the next, the overall dynamic range must remain unchanged. It is also critical to be sure that the sensor response does not depend on the amount expressed in the cell. There should not be a strong correlation between the response and the concentration of the sensor because a strong correlation can lead to misinterpretation of the data obtained. This is especially true for chemical sensors, while the expression of genetically encoded sensors is controlled in various ways.

## 6. Conclusions

The study of iPSCs has been one of the main directions in biology in recent decades. The properties of pluripotent cells provide abundant opportunities for their use in therapy and disease modeling, as well as in the search for and testing of new drugs. Currently, there are many well-developed methods for obtaining various types of differentiated cells from human iPSCs; researchers have developed technologies for creating organoids and tissues based on various iPSC derivatives. An enormous variety of cell models of human diseases, including neurodegenerative diseases such as Alzheimer’s disease, Parkinson’s disease, and other diseases, have been obtained based on iPSCs and their differentiated derivatives. The use of directed genome editing technology, as well as the use of chemical or genetically encoded biosensors in cell models, provides new knowledge about normal and pathological processes in living cells. To date, a massive variety of chemical biosensors have been developed, which make it possible to measure almost any process occurring in a living cell. However, the use of this type of biosensor has its drawbacks. There are commercial, chemically synthesized molecules that are not renewable and therefore must be purchased continuously. In addition, there is a problem with the delivery of chemical dyes to exact cellular compartments, which is solved by various modifications of the biosensor molecule. Complex modifications increase the size of the biosensor molecule, changing its physicochemical characteristics, which prevents its passage through the cell membrane. Another important point when using chemical biosensors is that the number of biosensor molecules entering the cell is unlimited. Therefore, if there is a correlation between the response and the biosensor concentration, the measurements obtained will not correspond to the actual picture occurring in the cell. Genetically encoded biosensors can be inserted into safe-harbor loci, for example, the *AAVS1* locus. It provides a strictly fixed number of transgene copies per genome, and its expression presumably occurs without disturbing the expression of adjacent or more distant genes. The use of an inducible promoter provides controlled expression, allowing the elimination of side and off-target effects. Using various combinations of genetically encoded biosensors embedded in several safe-harbor loci, or combining chemical biosensors with genetically encoded ones, one can thoroughly investigate the same process occurring in a cell. Even more remarkably, we can investigate several cellular processes at once, which allows us to gain more detailed knowledge of the events occurring in the cell in a healthy or pathological state. Today, the field of dynamic measurements of intracellular processes is developing very rapidly, and the leading task for the near future is the widespread use of the described technologies and methods not only in individual cells but also in more complex systems, such as cell organelles and whole organisms. The research of the mechanisms of complex biological processes, such as embryogenesis and aging, inflammation and regeneration, the functioning of tissues and organs in normal conditions and during the development of pathology, symbiotic interactions of organisms, the interplay between the host and the pathogen, and many others, requires in vivo models.

Low fluorescence intensity or small response amplitudes of biosensors can be practical limitations for in vivo use. Registration of subtle physiological changes in tissues with such instruments can become difficult. This is especially true for in vivo studies on mammalian models. As a rule, in these systems, the signal must be recorded in deep tissue structures, which requires special optical equipment.

Regardless of the biosensor used, it is important to remember that the introduction of an exogenous protein into the cell or organism can lead to unexpected effects that have an impact on physiological processes. Although it is generally accepted that fluorescent proteins are inert reporters, there are examples of their negative influence on intracellular processes in the literature [70].

Despite the above-mentioned possible limitations, biosensors have indisputable advantages over many other methods; they are gaining widespread popularity and are actively used for solving various questions, including drug screening, optimization, toxicity, or mechanism of action studies [71,72,73,74,75]. The development of biosensors for the detection of new compounds is a crucial line of future research that will open new perspectives for their application in experimental models [76,77,78,79,80,81].

A separate issue in the context of imaging with biosensors arises from the fact that shifts in the protein concentration or the thickness of the biological sample can result in signal alterations that might be taken for changes in the specific parameters. The described problem is especially relevant when imaging with single fluorescent protein-based sensors; however, FRET indicators are also prone to many artifacts. Common fluorescent proteins demonstrate relatively broad spectra, which result in tangible bleedthrough, making interpretation of ratiometric signals difficult. Moreover, ratiometric readout faces challenges in the case of confocal microscopy. Depending on the depth of the sample, patterns of light scattering for emission channels can differ noticeably, leading to measurement artifacts, especially in the case of in vivo imaging. A solution might be found in the implementation of FLIM readouts [82]. The main advantage of this approach is that fluorescence lifetime is a pure physical parameter independent of chromophore concentration, photobleaching, and the settings of equipment (intensity of excitation light and optical path).

Insufficient transparency of tissues for visible light due to various factors including melanin and hemoglobin absorbance and relatively pronounced scattering hamper imaging of multicellular organisms. Moreover, long microscopic series lead to a decrease in fluorescent biosensor brightness due to photobleaching. Widefield microscopy enhances this effect. Multiphoton microscopy methods can partially overcome these problems. This approach is based on simultaneous excitation of a chromophore by several photons with wavelengths which are longer than that of the emission maximum. Multiphoton microscopy also allows shifting of the source of excitation to the infrared region, which facilitates imaging of deep tissue regions. Since multiphoton absorption is characterized by low efficiency, this approach requires focusing the laser on a small sample volume, which reduces photobleaching and improves the signal-to-noise ratio. The improvement of biosensors, as well as the approaches for their visualization inside living organisms, will provide further progress for in vivo biomedical studies.

## Data Availability

Not applicable.
